# Primary Tumour Treatment in Stage 4 Colorectal Cancer with Unresectable Liver and Lung Metastases and No Peritoneal Carcinomatosis—Current Trends and Attitudes in the Absence of Clear Guidelines

**DOI:** 10.3390/jcm12103499

**Published:** 2023-05-16

**Authors:** Giovanni Domenico Tebala, Antonio Di Cintio, Francesco Ricci, Stefano Avenia, Roberto Cirocchi, Jacopo Desiderio, Domenico Di Nardo, Salomone Di Saverio, Alessandro Gemini, Maria Chiara Ranucci, Stefano Trastulli, Fabio Cianchi, Marco Scatizzi, Fausto Catena, the MeCC-4 International Collaborative

**Affiliations:** 1 Department of Digestive and Emergency Surgery, “S. Maria” Hospital Trust, 05100 Terni, Italy; 2 Department of General Surgery, “Madonna del Soccorso” Hospital, 63074 San Benedetto del Tronto, Italy; 3 Department of Digestive Surgery, “Careggi” University Hospital, 50134 Firenze, Italy; 4 Department of General Surgery, “S. Maria Annunziata e Serratori” Hospital, 50012 Firenze, Italy; 5 Department of General and Emergency Surgery, “Maurizio Bufalini” Hospital, 47521 Cesena, Italy

**Keywords:** colon cancer, primary tumour, liver metastases, lung metastases

## Abstract

Background: The treatment of the primary tumour in colorectal cancer with unresectable liver and/or lung metastases but no peritoneal carcinomatosis is still a matter of debate. In the absence of clear evidence and guidelines, our survey was aimed at obtaining a snapshot of the current attitudes and the rationales for the choice of offering resection of the primary tumour (RPT) despite the presence of untreatable metastases. Methods: An online survey was administered to medical professionals worldwide. The survey had three sections: (1) demographics of the respondent, (2) case scenarios and (3) general questions. For each respondent, an “elective resection score” and an “emergency resection score” were calculated as a percentage of the times he or she would offer RPT in the elective and in the emergency case scenarios. They were correlated to independent variables such as age, type of affiliation and specific workload. Results: Most respondents would offer palliative chemotherapy as the first choice in elective scenarios, while a more aggressive approach with RPT would be reserved for younger patients with good performance status and in emergency situations. Respondents younger than 50 years old and those with a specific workload of fewer than 40 cases of colorectal cancer per year tend to be more conservative. Conclusions: In the absence of clear guidelines and evidence, there is a lack of consensus on the treatment of the primary tumour in case of colon cancer with unresectable liver and/or lung metastases and no peritoneal carcinomatosis. Palliative chemotherapy seems to be the first option, but more consistent evidence is needed to guide this choice.

## 1. Introduction

Colorectal cancer (CRC) is one of the leading cancers worldwide and is responsible for more than 900,000 deaths every year [1]. Despite advancements in early diagnosis and prevention, a number of cases still arrive at a later stage at first presentation. About 20% of CRCs present as metastatic at the first diagnosis [2]. Furthermore, some patients with initially locally advanced CRC but without distant metastases at presentation can become metastatic during the neoadjuvant treatment.

Patients with infiltrated regional lymph nodes are not considered metastatic, but distant nodal involvement is considered to be metastatic. According to the TNM system [3], CRC with only regional nodal involvement can be staged within one of the subdivisions of Stage III, but in the presence of distant metastases the staging shifts to Stage 4. The most frequent sites of extranodal metastases are the liver, lungs and peritoneum. A case of metastases to only one site without peritoneal involvement is M1a (Stage IVa), while a case with metastases to two or more sites without peritoneal involvement is M1b (Stage IVb). Peritoneal metastases make the staging increase to M1c (Stage IVc), irrespective of other metastatic lesions. Clearly, the prognosis becomes poorer with increasing staging.

However, in the past few decades, the prognosis of patients with metastatic CRC has significantly improved with the development of lung and liver resective surgery in case of oligometastatic disease, but in the presence of extensive secondary lesions radical surgery is no longer an option and any treatment is only aimed at prolonging the survival and controlling the symptoms.

For elective patients, palliative chemotherapy can be effective, but the advantage it can offer in terms of overall survival is minimal and personalised effective treatments are yet to come [2].

Despite some interesting evidence showing that in selected cases resection of the primary tumour (RPT) can guarantee better survival than chemotherapy alone [4], palliative RPT is rarely offered to elective patients, being considered as a futile treatment [5]. RPT is more often offered in emergencies, more to control the acute complications than to pursue prolonged survival. On the contrary, RPT is not considered in the case of widespread peritoneal disease. However, the existing guidelines differ, and the therapeutic strategy is usually decided by the surgeon or the oncologist or, in the best-case scenario, the colorectal multidisciplinary team (CRMDT). With this survey, we aimed to obtain a snapshot of the current attitudes towards the treatment of the primary tumour in Stage IV CRC with unresectable liver and lung metastases and no peritoneal carcinomatosis (Stages IVa and IVb), with the hypotheses that (1) RPT is actually offered only to a small cohort of patients who may, on the contrary, benefit from increased survival should RPT be performed despite a clearly advanced disease and (2) there is lack of consensus and therefore high variability in the attitudes of doctors towards these patients.

## 2. Materials and Methods

An online survey was created using Google Forms (www.google.com/forms/about/ accessed on 16 March 2023). The survey was divided into 3 parts—Part 1: demographics of the respondent; Part 2: 12 clinical scenarios; and Part 3: general clinical questions. The “clinical scenarios” were 7 elective cases and 5 emergency cases. Although each of them can represent a real clinical situation, as all cases are quite general, none of the clinical scenarios were deliberately and overtly taken from experiences of real patients who came under our care and each reference to real persons is to be considered the full result of chance. Questions are reported in Table 1, Table 2 and Table 3 along with their responses. A link for the survey was created and shared through a professional social medium (www.linkedin.com accessed on 16 March 2023) and by email to the members of the Italian Association of Hospital Surgeons (ACOI = Associazione dei Chirurghi Ospedalieri Italiani), the Italian Society of Surgical Pathophysiology (SIFIPAC = Società Italiana di FisioPatologia Chirurgica) and the Tosco-Umbra Society of Surgery (Società Tosco-Umbra di Chirurgia). The link was also emailed to known colleagues in Italy and abroad.

It is not possible to specify how many doctors received the link for the survey, but we estimate the number should be no less than 2000. A completed survey was returned by 602 doctors, but only 508 agreed to join the MeCC-4 International Collaborative and can be listed as co-authors as they fulfilled the criteria of the International Committee of Medical Journal Editors [6]. Questionnaires with less than 70% of answers have been excluded.

The responses to the questionnaire were recorded in an electronic database (Microsoft Excel for Mac v.16.66.1, Redmond, WA, USA). The distribution of responses for every single question was calculated. Subsequently, individual scores were calculated for each respondent based on how often he or she would offer resection of the primary tumour according to the clinical scenarios, both in elective (“elective resection score”) and emergency situations (“emergency resection score”). The scores were calculated as a percentage of responses where “resection” was considered as the first choice on the total of elective or emergency scenarios. The scores are supposed to give an idea of the general attitude of that specific respondent. They were correlated to basic independent variables such as age, type of affiliation and specific workload. The variables were compared using a one-way ANOVA test (analysis of variance). All variables were entered into a backward stepwise regression analysis to identify the independent prognostic variables associated with the elective and emergency resection scores.

Statistics were performed within the same database with the add-on StatPlus for Mac v.7.8.11 (AnalystSoft Inc., Brandon, FL, USA). Missing values were excluded listwise. The demographics of the respondents are listed in Table 2. *p*-values less than 0.05 are considered to be statistically significant. Values of variables are approximated to the tenths. *p*-values are approximated to the thousandths.

## 3. Results

Table 1 reports the demographics of the respondents. Unfortunately, most respondents were men (79%), thus adding a possible bias. The vast majority of respondents were surgeons (98%).

Responses to the clinical questions (Section 2 and Section 3) are visualised in Table 2 and Table 3. The responses to the elective clinical scenarios showed that most respondents tend to offer chemotherapy as the first choice in patients with metastatic colon cancer and inoperable liver and/or lung metastases, reserving a more aggressive approach with RPT to younger patients with good performance status. Responses to the emergency clinical scenarios showed a more proactive attitude towards RPT.

**Figure 1:**
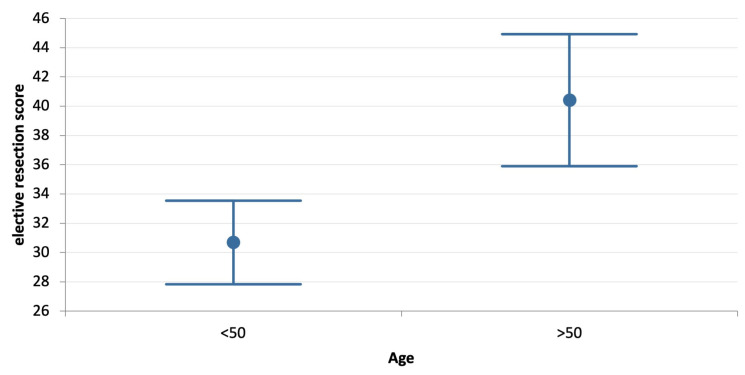
Comparison of “elective resection score” according to age (more or less than 50 years old) (*p* = 0.000).

**Figure 2:**
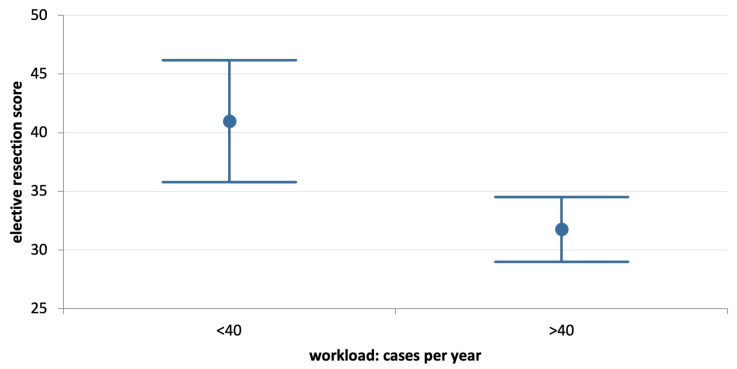
Comparison of “elective resection score” according to specific workload (more or less than 40 cases of colorectal cancers per year) (*p* = 0.001).

Table 4 reports the results of the comparative analysis of elective and emergency resection scores according to basic variables. The mean overall elective resection score is significantly lower than the emergency resection score (*p* = 0.000). Mean elective resection scores are also significantly lower than emergency resection scores for respondents who are younger and older than 50, for respondents who treat more than 40 colorectal cancers per year, for those affiliated with academic or non-academic hospitals, with or without a proper colorectal multidisciplinary team, and for consultants and non-consultants. Respondents younger than 50 years old have a significantly lower average elective resection score compared to more senior respondents (*p* = 0) (Table 4, Figure 1). This difference disappears in emergency scenarios (*p* = 0.645). Similarly, professionals who managed less than 40 cases of colorectal cancer per year have a higher elective resection score (*p* = 0.001) (Table 4, Figure 2). This difference disappears in emergency scenarios (*p* = 0.710). The elective resection score did not change significantly according to the type of practice.

Due to the fact that none of the analysed variables resulted significantly associated with the emergency resection scores, regression analysis was conducted only for elective resections scores (Table 5), and it confirmed that age and workload are independent prognostic variables, with older age and lower workload being associated with higher elective resection score.

## 4. Discussion

Advanced colorectal cancer with non-treatable distant metastases is associated with poor prognosis. Palliative chemotherapy can help prolong survival, but the advantage brought by chemotherapy alone is only marginal, despite huge improvements in targeted and personalised treatments [2]. Bearing in mind that metastatic colorectal cancer is only potentially treatable if the metastatic burden is radically resectable along with the primary tumour, patients with unresectable metastases can only be offered palliative treatment to prolong survival and control the symptoms. Resecting the primary tumour while leaving alone unresectable liver and lung metastases can be a debatable option. Some evidence seems to suggest that cytoreduction by RPT may offer a significant improvement in survival [4], as long as the surgical risk is low and the operation does not excessively delay the start of chemotherapy, but this has never been definitely confirmed. The decision to offer RPT, or against this option, is based on unclear lines of reasoning.

After denying any advantage of RPT for many years and suggesting upfront chemotherapy in all patients with Stage IV CRC, the last edition of the UK NICE (National Institute for Health and Care Excellence) guidelines on colorectal cancer clearly suggest that RPT should be considered in patients with “incurable metastatic colorectal cancer who are receiving systemic anti-cancer therapy and have an asymptomatic primary tumour” [7] on the grounds that RPT can prolong survival and avoid symptoms related to the primary tumour, such as obstruction, perforation and bleeding. According to the NICE guidelines, RPT is associated with a low risk of complication (5%), while upfront palliative chemotherapy is associated with a 20% risk of primary tumour-related symptoms needing treatment at some point during the clinical course of these unfortunate patients.

The guidelines of the Association of Coloproctology of Great Britain and Ireland (ACPGBI), those of the European Society for Medical Oncology (ESMO) and those of the German Guideline Program in Oncology do not mention the possibility of RPT and consider upfront chemotherapy in these cases [8,9,10].

The US National Comprehensive Cancer Network (NCCN) guidelines and those of the American Society of Colon and Rectal Surgeons (ASCRS) suggest upfront systemic chemotherapy and consider RPT only in case of significant symptoms or complications [11,12].

The 2020 guidelines of the Italian Association of Medical Oncology (AIOM) almost overlap with the NCCN and the ASCRS guidelines and briefly suggest RPT if the primary tumour is symptomatic, but without discussing the available evidence [13].

Evidently, there is a degree of variability among the several national and international guidelines, reflecting slightly different points of view.

The JCOG1007 study from Japan published in 2021 was terminated early due to futility as the first interim analysis showed that the predictive probability of survival being higher in the RPT group than in the chemotherapy group would be quite low at the final analysis if the study were to be continued. In fact, the updated final analysis on 165 patients failed to demonstrate better survival in the RPT group compared to the chemotherapy group [14].

A specific randomized controlled trial comparing RPT to upfront chemotherapy in metastatic CRC, with a median follow-up of 15 months, was recently conducted in South Korea [4]. The study had quite a small sample size (52 patients, 27 allocated to RPT + chemotherapy and 25 allocated to upfront chemotherapy, but only 23 + 21 = 44 were analysed) and the randomisation process is not clear. These important flaws notwithstanding, the results are quite interesting, as they showed a significant improvement in cancer-specific 2-year survival along with a non-statistically significant overall 2-year survival (not reaching statistical significance due to the small sample size) with RPT + chemotherapy compared to chemotherapy alone [4].

The CAIRO4 phase 3 randomized controlled trial focused on 60-day mortality of patients randomized to RPT vs. upfront chemotherapy, and showed that RPT is associated with a higher risk of mortality compared to chemotherapy alone [15], in particular in patients in poorer general conditions. However, the analysis of the causes of death showed that only one patient in the RPT group died of surgical complications, whereas all the others died either of disease progression, toxicity of the systemic treatment or other causes not related to treatment. It is worth highlighting that the CAIRO4 study involved only patients with no symptoms from the primary tumour, and therefore resection was not aimed at controlling symptoms or treating a complication. The CAIRO4 trial was designed mostly to quantify the surgical risk of RPT and to clarify its futility but does not give any indication of the long-term benefit of RPT.

The FFCD 9601 study proved that the real advantage of RPT vs. upfront chemotherapy is in prolonging survival. In fact, median survival was 16.3 vs. 9.5 months, 2-year survival was 24% vs. 10% and 6-month progression-free survival was 38% vs. 22%. All these results were statistically significant. In multivariate analysis, RPT was the strongest independent factor associated with improved survival. Good performance status and distal location of the tumour were also independently associated with better survival. In other terms, good results in terms of improved survival could be obtained in patients in good general condition undergoing resection of a distal colonic primary tumour even in the presence of unresectable distant metastases [16].

An 11-year-old metanalysis by our team of seven low-quality studies involving 1086 pooled patients failed to find any survival benefit in RPT compared to upfront chemotherapy [17], but a more recent metanalysis from China on 8 studies involving 2805 patients disproved those results and reported significantly better 2-year, 3-year and 5-year survival with RPT compared to chemotherapy [18]. This more recent paper is based on good quality studies including three randomised controlled trials, and therefore we tend to consider its findings more reliable.

A pooled post hoc analysis performed on 1155 cases from four trials showed that RPT guarantees better overall survival compared to chemotherapy alone in patients with unresectable metastatic CRC [19]. As in most of the other studies, in this large analysis colon and rectal cancers have also been mixed, creating a possible selection bias.

On the contrary, a large observational retrospective cohort study from the US on 6735 patients from the National Cancer Data Base concluded that RPT does not improve survival and may delay the onset of palliative chemotherapy. However, despite the large sample size, the study has multiple limitations, including some degree of selection bias (excluding patients who were eventually treated with conversion chemotherapy with curative intent and including those who had poorer prognoses) [20].

Xu et al. retrieved data from the US National Cancer Institute’s Surveillance, Epidemiology and End-Results database, covering about 30% of the US population, and identified 44,514 patients with stage IVa and IVb CRC. Survival gain for patients who had RPT was 7–11 months (median 9 months) compared to those who have been treated only with any form of chemotherapy (median survival 16 months). RPT was independently related to better survival upon multivariate analysis. Furthermore, RPT patients had also a significantly lower likelihood of all-cause death [21].

Other published papers are low or very low-quality retrospective cohort studies, with most of them showing that RPT is associated with better 2-year survival [22].

Finally, the SYNCHRONOUS study was launched in 2012 and aimed at comparing RPT and chemotherapy in asymptomatic Stage IV CRC patients. Its main endpoint is long-term survival, but its results are still pending [23].

Guidelines and evidence do not fully agree on this subject, and the decision to offer RPT or not is still up to the surgeon, the oncologist and the CRMDT, if present, but unsupported by clear guidance.

This study was aimed at obtaining a snapshot of the current attitudes of medical professionals on the treatment of primary tumours in Stage IVa and IVb CRC.

Our survey showed that the vast majority of professionals base their decision on age, comorbidities, performance status and symptoms. In fact, about half of the respondents to our survey would consider RPT in patients who presented as emergencies against only one-third who would consider RPT in elective and non-symptomatic patients.

Only one-third of the respondents seem to be so confident as to base their decision on the K-RAS status. This finding might have something to do with the fact that most respondents are surgeons, who may not have the knowledge to understand how a naïve or mutant K-RAS gene can influence the therapeutic options. As a matter of fact, the presence of a naïve K-RAS would allow the oncologist to consider second- or third-line chemotherapy with monoclonal antibodies, while patients with mutant K-RAS (or N-RAS or BRAF) may have fewer options available, so cytoreductive surgery may become the last resort.

Younger respondents seem to have a less aggressive approach in elective cases. This is quite surprising. A possible explanation could be that more experienced doctors have a more patient-centred attitude, trying to do the maximum for that patient despite all odds, against a more evidence-centred attitude of younger doctors who may be a little more realistic, considering that those patients have a poor prognosis anyway.

Much more understandable is the finding that respondents with lower workloads—in terms of the number of CRCs treated per year—would offer RPT more often, to try and increase their surgical experience.

These two variables—age and workload—also resulted significantly and independently correlated with the elective resection score upon multivariate analysis, thus confirming the findings of the univariate analysis.

The nature of the hospital, the presence of a colorectal multidisciplinary team and professional seniority did not influence the choice to offer resection or not, both in elective and emergency scenarios.

The main limitation of this study is the uneven geographical distribution of respondents, with most of them being from Southern Europe (75%), the unbalanced gender distribution, with most of the respondents being men (79%), and the prevalence of surgeons among the respondents (98%). It is not clear how much the results could have been biased because of this unequal distribution. However, we feel that a basis of more than 600 respondents is in any case a good sample size and may fairly represent the general attitude towards a topic that has never been clearly standardised and is probably not fully standardisable anyway. Another eventual downside of this survey is that the 12 clinical scenarios do not cover all the possible situations, and there are still grey areas that have not been explored by the survey. While we can appreciate that this may be a minor issue, we must emphasise that adding more and more questions would have made the survey hardly acceptable to potential respondents. Furthermore, we feel that the depicted clinical scenarios give a very good idea of the general attitude of the medical community towards the delicate topic of the treatment of the primary lesion in metastatic colorectal cancers.

The lack of high-level evidence and specific evidence-based guidelines makes the indication of RPT in these patients still a matter of debate. A proper long-term multicentric randomized clinical trial with a large sample would hopefully be able to clarify this topic and shed some light on the decision-making for these patients.

In conclusion, this survey has demonstrated that doubts remain in both elective and emergency situations on the treatment of the primary tumour in Stage 4 CRC with non-operable liver and/or lung metastases but no peritoneal carcinomatosis. Palliative chemotherapy seems to be the first option, particularly in elective situations, but more consistent evidence is much needed to guide this choice.

## Figures and Tables

**Table 1:** Responses to Section 1. Demographics of the respondents.

Factor	Number	%
*Total*	602	100
*Gender*		
Men	476	79.1
Women	124	20.6
Other/Does not respond	2	0.3
*Age*		
<30	21	3.5
30–40	239	39.7
40–50	137	22.8
50–60	125	20.8
>60	80	13.3
*Degree of experience*		
Trainee	57	9.5
Registrar/Senior Trainee/SAS doctor	100	16.6
Consultant	368	61.1
Other	75	12.5
Missing	2	0.3
*Specialty*		
General Surgery	505	83.9
Colorectal Surgery	79	13.1
Upper GI Surgery	5	0.8
Medical Oncology	4	0.7
Clinical Oncology/Radiotherapy	6	1.0
Other	3	0.5
*Main place of work*		
University Hospital	224	37.2
Teaching Hospital	79	13.1
District General Hospital	190	31.6
Community Hospital	68	11.3
Private Hospital	33	5.5
Private Practice/Clinic	1	0.2
Other	7	1.2
*Zone*		
Northern Europe	28	4.7
Continental Europe	88	14.6
Southern Europe	449	74.6
Eastern Europe	14	2.3
USA/Canada	2	0.3
Central America	0	0.0
South America	3	0.5
North Africa	3	0.5
Central Africa	2	0.3
South Africa	1	0.2
Near East	0	0.0
Middle East	5	0.8
Far East/Asia	2	0.3
Oceania	0	0.0
Other/Does not respond	5	0.8
*Is there a regular Colorectal Cancer MDT in your hospital?*		
Yes	542	90.0
No	60	10.0
*How many colorectal cancers you see/treat in 1 year?*		
<20	40	6.6
20–40	106	17.6
40–60	129	21.4
60–80	87	14.5
80–100	97	16.1
>100	142	23.6
Does not respond	1	0.2

**Table 2:** Responses to Section 2. Questions on clinical cases. Options with zero preferences have been omitted.

Clinical Cases	Treatment	N.	%
Case 1. Patient with asymptomatic Stage IV left colon cancer with inoperable liver metastases; no other metastases; age 43; ASA 2; WHO Perf. 0; and K-RAS naive.	Resection of the primary tumour + chemotherapy	284	47.2%
	Chemotherapy	309	51.3%
	End-of-life care	1	0.2%
	Other	8	1.3%
	Total	602	100%
Case 2. Patient with asymptomatic Stage IV sigmoid colon cancer with inoperable liver metastases; no other metastases; age 82; ASA 2; WHO Perf. 0; and K-RAS mutant.	Resection of the primary tumour + chemotherapy	186	30.9%
	Chemotherapy	315	52.3%
	Surveillance	40	6.6%
	End-of-life care	44	7.3%
	Other	17	2.8%
	Total	602	100%
Case 3. Patient with asymptomatic Stage IV right colon cancer with inoperable liver metastases; no other metastases; age 67; ASA 4; WHO Perf. 3; and K-RAS naive.	Resection of the primary tumour + chemotherapy	117	19.4%
	Chemotherapy	336	55.8%
	End-of-life care	123	20.4%
	Other	26	4.3%
	Total	602	100%
Case 4. Patient with asymptomatic Stage IV rectal cancer with inoperable liver and lung metastases; age 72; ASA 3; WHO Perf. 2; and K-RAS mutant.	Resection of the primary tumour + chemotherapy	69	11.5%
	Chemotherapy	371	61.6%
	Radiotherapy	89	14.8%
	Surveillance	15	2.5%
	End-of-life care	25	4.2%
	Other	33	5.5%
	Total	602	100.%
Case 5. Patient with perforated Stage IV sigmoid tumour with inoperable liver and lung metastases; age 65; ASA 1; and WHO Perf. 0.	Emergency resection of the primary tumour + chemotherapy	455	75.6%
	Emergency resection of the primary tumour + surveillance/end-of-life care	17	2.8%
	Emergency drainage + ileostomy/colostomy + elective resection of the primary tumour + chemotherapy	60	10.0%
	Emergency drainage + ileostomy/colostomy + chemotherapy	64	10.6%
	Other	6	1.0%
	Total	602	100%
Case 6. Patient with obstruction due to Stage IV right colon cancer with inoperable liver and lung metastases and ascites; age 54; ASA 2; and WHO Perf. 2.	Emergency resection of the primary tumour + chemotherapy	265	44.0%
	Emergency ileostomy/caecostomy + elective resection of the primary tumour + chemotherapy	92	15.3%
	Emergency ileostomy/caecostomy + chemotherapy	117	19.4%
	Stent + chemotherapy	110	18.3%
	Stent + surveillance	12	2.0%
	End-of-life care	2	0.3%
	Other	4	0.7%
	Total	602	100%
Case 7. Patient with severe acute anaemia and rectal bleeding; cancer of the caecum with inoperable liver and lung metastases; age 70; ASA 2; and WHO Perf. 1.	Emergency resection of the primary tumour + chemotherapy	247	41.0%
	Embolization + chemotherapy	41	6.8%
	Embolization + elective resection of the primary tumour + chemotherapy	57	9.5%
	Transfusions + chemotherapy	18	3.0%
	Transfusions + elective resection of the primary tumour + chemotherapy	229	38.0%
	Transfusions + surveillance	6	1.0%
	Other	4	0.7%
	Total	602	100%
Case 8. Patient with asymptomatic Stage IV right colon cancer with inoperable lung metastases and ascites; age 47; ASA 3; and WHO Perf. 3.	Resection of the primary tumour + chemotherapy	153	25.4%
	Chemotherapy	392	65.1%
	Surveillance	34	5.6%
	Other	23	3.8%
	Total	602	100%
Case 9. Patient with asymptomatic Stage IV distal transverse colon cancer with inoperable liver metastases; age 40; ASA 1; WHO Perf. 0; and K-RAS naive.	Resection of the primary tumour + chemotherapy	311	51.7%
	Chemotherapy	283	47.%
	Surveillance	2	0.3%
	Other	6	1.%
	Total	602	100%
Case 10. Patient with asymptomatic Stage IV cancer of the proximal transverse colon with inoperable liver metastases; age 55; ASA 1; WHO Perf. 0; and K-RAS mutant.	Resection of the primary tumour + chemotherapy	313	52.%
	Chemotherapy	272	45.2%
	Surveillance	4	0.7%
	Other	13	2.2%
	Total	602	100%
Case 11. Patient with obstructing Stage IV cancer of the splenic flexure, ascites and inoperable lung and liver metastases; age 65; ASA 4; and WHO Perf. 4.	Emergency resection of the primary tumour + chemotherapy	109	18.1%
	Emergency ileostomy/colostomy + chemotherapy	230	38.2%
	Emergency ileostomy/colostomy + elective resection of the primary + chemotherapy	54	9.%
	Emergency ileostomy/colostomy + surveillance	129	21.4%
	End-of-life care	53	8.8%
	Other	27	4.5%
	Total	602	100%
Case 12. Patient with severe anaemia due to bleeding rectal cancer with inoperable liver and lung metastases; age 67; ASA 1; and WHO Perf. 1.	Emergency resection of the primary tumour + chemotherapy	98	16.3%
	Embolization/endoscopic haemostasis + elective resection of the primary + chemotherapy	116	19.3%
	Embolization/endoscopic haemostasis + chemotherapy	187	31.1%
	Transfusions + elective resection of the primary + chemotherapy	132	21.9%
	Transfusions + chemotherapy	34	5.6%
	End-of-life care	2	0.3%
	Other	33	5.5%
	Total	602	100%

**Table 3:** Responses to Section 3. General clinical questions.

What factors do you consider as priority in the decision-making process in a case of a Stage IV colorectal cancer with inoperable liver and lung metastases? (multiple choice)	Age	444	73.8
	Emergency presentation	442	73.4
	Symptoms	423	70.3
	ASA class	421	69.9
	Presence of ascites/carcinomatosis	396	65.8
	WHO Performance status	365	60.6
	Guidelines	305	50.7
	K-RAS status	202	33.6
	Number of metastatic sites	177	29.4
	Preference of the patient	144	23.9
	Other	63	10.5
	Availability of a skilled colorectal surgeon	52	8.6
	Local availability of chemotherapy facilities	52	8.6
	Local availability of biologics/third-line chemotherapy	37	6.1
	Cost/Financial implications	9	1.5
Do you regularly offer/consider resection of the primary tumour in Stage IV colorectal cancer patients with inoperable liver and lung metastases?	Always	19	3.2%
	Often	155	25.7%
	Sometimes	262	43.5%
	Rarely	142	23.6%
	No	23	3.8%
	Missed/Does not answer	1	0.2%

**Table 4:** Comparative analysis of elective and emergency resection scores according to basic variables (RS = resection score; yo = years old). In bold: significant *p*-values.

Variables		n.	Elective RS	Emergency RS	*p*
Total		602	34.0 ± 30.6	45.8 ± 17.1	***0.000***
Age	<50 yo	397	30.7 ± 28.9	45.6 ± 16.8	***0.000***
	>50 yo	205	40.4 ± 32.8	46.3 ± 17.6	***0.024***
	*p*		***0.000***	*0.645*	
Workload	<40 CRC/year	146	41.0 ± 31.7	46.3 ± 18.3	*0.081*
	>40 CRC/year	456	31.8 ± 30.0	45.7 ± 16.7	***0.000***
	*p*		***0.001***	*0.710*	
Affiliation	Academic	303	33.4 ± 30.9	46.8 ± 17.1	***0.000***
	Non-academic	258	33.9 ± 30.7	45.0 ± 17.2	***0.000***
	Private/Other	41	39.4 ± 28.5	44.3 ± 16.8	*0.345*
	*p*		*0.500*	*0.382*	
Seniority	Consultant	368	32.0 ± 31.0	45.9 ± 17.0	***0.000***
	Non-consultant	234	35.7 ± 30.0	45.7 ± 17.3	***0.000***
	*p*		*0.275*	*0.857*	
Colorectal cancer MDT	Yes	542	33.9 ± 30.9	45.9 ± 16.9	***0.000***
	No	60	35.2 ± 27.8	45.2 ± 19.0	***0.023***
	*p*		*0.743*	*0.780*	

**Table 5:** Backward stepwise regression analysis of elective resection scores. Model fitness: R = 0.189, R2 = 0.036; *p* = 0.000; yo = years old.

Significant Variables	Coefficient	*p*
Age (>50 yo vs. <50 yo)	9.0	0.001
Workload (>40/y vs. <40/y)	−8.2	0.004
Intercept	37.2	

## Data Availability

The datasets generated and analysed in the present study are available from the corresponding author upon reasonable request.

## Appendix A

The Metastatic Colorectal Cancer Stage 4 (MeCC-4) International Collaborative:

Albania: Braholli E (Dibër), Agastra E (Korçe). Algeria: Bouzid C (Algiers), Fillali T (Aokas). Argentina: Nari G (Córdoba). Azerbaijan Samadov E (Baku). Djibouti Venezia P (Djibouti). Greece: Korkolis DP (Athens), Anestiadou E, Orestis I (Thessaloniki). India: Mehraj A (Srinagar). Iran: Saeidi S (Mashhad). Italy: Sergio G (Acerra), Targa S (Adria), Gentili I (Albano), Carboni L (Alghero), Ialongo P (Altamura), Maurizi A (Ancona), Cianci P, Restini E (Andria), Brachet Contul R, Grivon M, Usai A (Aosta), Sulce R (Arezzo), Guercioni GL (Ascoli Piceno), Liberatore E (Atri), Di Nardo C, Landolfi V (Avellino), Marra E (Aversa), De Luca R, Lomonaco R (Bari, Ist.Tumori), Lafranceschina S (Bari, Osp. S.Paolo), Papagni V (Bari, Osp.”Di Venere”), Martines G, Giove C, Negro G, Pasculli A, Piccinni G, Trigiante G (Bari, Università), Sinisi G (Barletta), Argenio G (Benevento), Poletti E, Stefano M (Bergamo, Osp.Papa Giovanni XXIII), Pinotti E (Bergamo, Pol.S.Pietro), Cammillini F (Bibbiena), Polastri R (Biella), Caraglia A (Bisceglie), Mastrangelo L, Raspanti A, Zanello M (Bologna, Osp. Maggiore), Rottoli M, Russo I, Torre B, Tosi L, Zanotti S (Bologna, S. Orsola), Frena A, Marinello P (Bolzano), Bono D (Borgosesia), Andreuccetti J, De Capua M (Brescia, Spedali Civili), Baiocchi GL, Portolani N, Tiberio G (Brescia, Università), Calò G (Brindisi), Roscio F (Busto Arsizio (MI)), Carrano F (Busto Arsizio (VA)), Manunza R, Runfola M (Cagliari, Brotzu), Cillara N (Cagliari, SS Trinità), Podda M (Cagliari, Università), Locurto P (Caltanissetta), Casati M (Carate Brianza), Cravero F (Casale Monferrato), Andreano M, Scala D (Caserta), Marino F (Castellana Grotte), Traficante A (Castelvetrano (TP)), Cinardi N, Evola G (Catania, Garibaldi), Veroux M (Catania, Pol.S.Marco), Leone E, Zerbo D (Catania, Policlinico), Ammendola M, Cardona R, Curro G, Rizzuto A, Romano R, Sena G (Catanzaro), Albanese F (Cerignola), Bertelli R, Catena F (Cesena), Cappato S (Chiavari), Barone M, Gargarella S, Mucilli F (Chieti), Carrera M (Ciriè), Portale G (Cittadella), Florio G (Colleferro), Cantore F (Como), Pavanello M (Conegliano), Chiarello MM, La Gumina G, Nardo B (Cosenza), Celotti A, Ranieri V, Rovatti M, Somenzi D (Cremona), Cannistrà M, Castaldo P (Crotone), Giraudo G, Giuffrida M (Cuneo), Maggioni D, Gerosa M (Desio), Sparavigna L (Eboli), Calistri M, Di Mare G (Empoli), Centonze D, Licciardello A (Enna), Taglietti L (Esine), Budassi A (Fabriano), Di Candido F (Faenza), Guerriero S (Fermo), Anania G (Ferrara), Alemanno G, Bencini L, Bottari A, Cianchi F, Fortuna L, Prosperi P, Scheiterle M (Firenze, Careggi), Scatizzi M (Firenze, S.Maria Annunziata), Giuliani A, Lizzi V (Foggia, Policlinico), Pacilli M (Foggia, Università), Ceccarelli G (Foligno), Lucci E, Pacilio C, Ercolani G (Forlí), Bellanova G (Francavilla Fontana), Corelli S, Di Cello PF (Frosinone), Cestaro G (Gallarate), Merlini D (Garbagnate), Fontana T (Gela), Oliva A (Genova, Evangelico), Azzinnaro A, Barberis A, Razzore A (Genova, Galliera), Amisano M, Luzzi A, Pertile D, Santoliquido M, Scabini S (Genova, S.Martino), Ribeca U (Genova, Villa Scassi), Rizza V (Giulianova), Benigni R, Giuliani G (Grosseto), Autuori F (Iglesias), Amato A (Imperia), Clementi M (L’Aquila), Denise G (Lamezia Terme), Ceci F, Greco L (Latina), Muzio E (Lavagna), Libia A, Spampinato M (Lecce), Malagnino A, Zago M (Lecco), Spalluto M (Legnano), Galatioto C (Livorno), Bisagni P (Lodi), Castiglioni S (Macerata), Siquini W (Macerata), Bertoglio CL, Dinuzzi V (Magenta), Aldighieri F, Farfaglia R (Manerbio), Brandimarte A, Mantovani G (Mantova), Guaitoli E, Perrone F (Martina Franca), Comandè M, Magistro C (Melegnano), Baldini E (Melzo), Fleres F, Saladino E (Messina), Barbaro S, Rivolta U (Milano, ASST Ovest), Galfrascoli E, Maffioli A, Mazzotta E (Milano, FBF), Aiolfi A, Manara M (Milano, Galeazzi), Milana F (Milano, Humanitas), Pizzini P (Milano, IEO), Ferrario L (Milano, INT), Lo Conte D, Soldini G (Milano, Multimedica), Andrea B, Franchi E, Iacob G, Kurihara H, Scaravilli L (Milano, Policlinico), Balla A (Milano, S.Raffaele), Formisano G, Mariani N, Zaccone S (Milano, SS Paolo e Carlo), Caponnetto A (Militello-Val di Catania), Verdi D (Mirano), Ascari F, Casoni Pattacini G, Trapani V (Modena), Esposito S, Pecchini F (Modena, Baggiovara), Ceresoli M, Chimenti F, Ciulli C, Degrate L, Golia M, Totis M (Monza), Antropoli C, Brillantino A, Di Martino M, Grillo M, Neola B, Pisaniello D, Vennarecci G (Napoli, Cardarelli), Amato B, Anoldo P, Basile R, Capuano M, Cricrì M, Giovanni A, Palomba G, Peltrini R, Schiavone V (Napoli, Federico II), Barra L (Napoli, Monaldi), Andrea T, Armellino MF, Falco P, Guida F, Marte G (Napoli, Osp.del Mare), Bottino V (Napoli, Osp.Evangelico Betania), Belli A, Pace U (Napoli, Pascale), Pizza F (Napoli, Rizzoli), Formisano V (Napoli, S.Giovanni Bosco), Menna MP, Pellino G (Napoli, Vanvitelli), Barugola G (Negrar di Valpolicella), Bufalari A (Nottola), Corlianó A, Monni M, Romito R (Novara), Basile E (Orvieto), Camperchioli I (Ostia), Armellin C, Bellio G, Moletta L, Pierobon ES, Schiavon N (Padova), Fabozzi M (Pagani), Martorana G (Palermo, FBF), Speciale A (Palermo, Orestano), Spinnato G (Palermo, Osp.”G.F.Ingrassia”), Carpino S, Frazzetta G, Morfino G (Palermo, Osp.Civico), Branca M, Lupo M, Mirabella A (Palermo, Villa Sofia-Cervello), Sorrentino M (Palmanova), Annicchiarico A, Dalmonte G, Giuffrida M (Parma), Dominioni T, Fugazzola P, Martinotti M, Trotta F (Pavia), Arcuri G (Perugia), Coletta D, Patriti A (Pesaro), Feroci F (Pescia), Capelli P, Piccolo D (Piacenza), De Zuanni M, Muratore A (Pinerolo), Barbato G, Poli G (Piombino), Cecconi C (Piove Di Sacco), Buccianti P, Chiarugi M, Coccolini F (Pisa), Giannessi S, Monati E (Pistoia), Montuori M (Ponte S.Pietro), Roveda L (Pontedera), Ubiali P (Pordenone), Del Vecchio G (Potenza), Mauriello C, Pirozzi F (Pozzuoli), Corsale I, Giordano A (Prato), Giacometti M (Reggio Emilia), Garulli G (Riccione), Parlanti D, Togni C (Rimini), Caputo D, Cammarata R, Carannante F, Cascone C, D’Ercole G, Farolfi T, Fiore M, La Vaccara V, Marco C, Petrianni G (Roma, CBM), Catarci M (Roma, Pertini), Garcea A (Roma, Pol.Casilino), Angelico R, Antonelli A, Bellato V, Flaminio V, Franceschilli M, Petagna L, Pirozzi B, Sica G (Roma, PTV), Cardella S, Lo Dico R, Ricci G (Roma, S.Camillo-Forlanini), Carlini M, Grieco M, Lisi G, Spoletini D (Roma, S.Eugenio), Cordiva Herencia I, Mazzarella G, Oricchio D, Rosa A, Rossi M, Rossi S, Solinas L (Roma, S.Filippo Neri), Falbo F, Fiori G, Pende V (Roma, S.Giovanni-Addolorata), Di Paola M, Gazia C (Roma, S.Pietro), Lepre L (Roma, S.Spirito), Caronna R, Cicerchia P, Coppola A, Corallino D, D’Ambrosio G, Ferent I, Fiori E, Gallo G, Iannone I, Iossa A, Lucchese S, Meneghini S, Mingoli A, Mongardini M, Pace M, Paradiso G, Perfetto F, Quaresima S, Rinaldi V, Saullo P, Sbacco V, Usai S, Zambon M (Roma, Sapienza), Bianchi V, Brisinda G, Brisinda G, D’Ugo D, Ferri L, Fico V, Fransvea P, Galiandro F, Giambusso M, Giovinazzo F, La Greca A, Lodoli C, Lorenzon L, Puccioni C, Rosa F, Schena CA, Tropeano G (Roma, UCSC), Pata F (Rossano), Scudo G (Rovereto), Parini D, Romeo F, Zese M (Rovigo), Bazzocchi F, Ricciardiello M (S.Giovanni Rotondo), Muto C (S.Maria CV), Calabrese P, Donnarumma E, Pilone V, Saviello C (Salerno), Petitti T (San Severo), Andolfi E (Sansepolcro), Delogu D, Fais E, Vargiu I (Sassari, Ozieri), Puledda M (Sassari, Policlinico Sassarese), Barmina M, Mucci G, Perra T, Porcu A, Porzani S, Scanu A (Sassari, Università), Malerba M (Savona), Maglio R (Scorrano), Costanzo A (Seriate), Mariani F (Siena), Trovatello A (Siracusa), Clarizia G, Spolini A (Sondrio), Poillucci G (Spoleto), Sacco L (Teramo), Vallo A (Termoli), Avenia S, Cirocchi R, Desiderio J, Di Cintio A, Di Nardo D, Farinacci F, Garofoli E, Gemini A, Guerci L, Mazzetti S, Napolitano V, Pennetti Pennella F, Ranucci MC, Ricci F, Spizzirri A, Tebala GD, Trastulli S, Trippa F (Terni), Borghi F (Torino, Candiolo), Borasi A, Ossola P (Torino, Humanitas Gradenigo), Borreca D (Torino, Martini), Fazio F, Zingaretti C (Torino, Mauriziano), Arezzo A, Comba A, Deiro G, Marano A, Moro F, Santarelli M, Tancredi M (Torino, Molinette), Bellocchia AB (Torino, Osp.Maria Vittoria), Giovanni G, Muzio M (Trapani), Brolese A, Crepaz L, Motter M (Trento), Brizzolari M, Romano M, Sartori A (Treviso), Mita MT (Tricase), Biloslavo A, Casagranda B (Trieste), Cojutti A, Mozzon M (Udine), Desio M, Lauro R (Varese), Platto M (Varese, Humanitas Mater Domini), Arroyo-Murillo G, Mondi I (Venezia), Daffara M (Vercelli), d’Addetta MV, Pedrazzani C, Turri G, Valdegamberi A, Zigiotto D (Verona), Talarico C (Vibo Valentia), Poli F, Zaghi C (Vicenza), Damiani G (Vigevano), Cotsoglou C, Granieri S (Vimercate), Amodio P (Viterbo), Ragazzi S (Vittoria), Olmi S (Zingonia). Luxembourg: Fassari A (Luxembourg). Morocco: Amine S (Rabat). Poland: Pach R (Cracow). Qatar: Kurer M (Doha). Romania: Dumbrava B, Gorgan CL, Grama F, Octavian E, Parvuletu R, Toma E, Valentin C (Bucharest), Martiniuc A (Buzesti). South African Republic: Marais P (Morningside, Johannesburg). Switzerland: Mayer J (Geneva). UAE: Azfr M (Abu Dhabi). United Kingdom: Peravali R (Birmingham), Justin D (Cambridge), Murphy J, Salem A (London). United States of America: Sciortino T (New York).
